# Parrot politics: social decision-making in wild parrots relies on both individual recognition and intrinsic markers

**DOI:** 10.1098/rsos.241542

**Published:** 2025-05-28

**Authors:** Julia Penndorf, Damien R. Farine, John M. Martin, Lucy M. Aplin

**Affiliations:** ^1^ Max Planck Institute of Animal Behavior, Radolfzell, Baden-Württemberg, Germany; ^2^ Division of Ecology and Evolution, Research School of Biology, Australian National University, Canberra, Australian Capital Territory, Australia; ^3^ Department of Evolutionary Biology and Environmental Studies, University of Zurich, Zurich, Switzerland; ^4^ Department of Collective Behavior, Max Planck Institute of Animal Behavior, Radolfzell, Germany; ^5^ Western Sydney University Hawkesbury Institute for the Environment, Penrith, New South Wales, Australia

**Keywords:** social cognition, social hierarchies, fission–fusion dynamics, social heuristics, aggressive interactions, Psittaciformes

## Abstract

Dominance hierarchies are generally thought to form over time via memory of repeated interactions. Yet dominance hierarchies are also occasionally reported in species with fission–fusion social dynamics, where individuals may encounter large numbers of individuals, leading to incomplete social information. In these cases, three potential mechanisms have been proposed. First, the complex decision-making required could increase selection for social cognition. Second, so-called ‘badge-of-status’ could evolve as shortcuts. Third, mixed strategies could evolve that rely on memory for interactions with familiars and status signals for strangers. Here, we test these hypotheses in wild sulphur-crested cockatoos (*Cacatua galerita*), recording social associations and aggressive interactions of 411 individuals across three neighbouring roosts. We find cockatoos use a twofold social strategy when initiating or reacting to aggression. For familiar individuals, aggressions were initiated or responded to based on differences in dominance rank. However, when facing less familiar individuals, decisions to interact—or respond—were based on body weight, with interactions directed towards, and more likely to respond to, individuals of similar weight. Our results suggest that social knowledge remains an important determinant of aggressive interactions in fission–fusion systems, but that individuals can dynamically incorporate other cues of competitive ability when knowledge is lacking.

## 1. Introduction

Contests over limited resources are a fact of life for almost all social species [1] and can potentially be extremely costly for the animals involved. Various mechanisms should therefore evolve that allow animals to better assess competitors and more accurately target a subset of individuals to engage with, decreasing the overall level of aggression [2,3]. There have been two main mechanisms proposed. The first is the so-called *badge-of-status* (first described by [4], later formalized by [5]; by [6]). Here, individuals exhibit honest signals that correlate with fighting ability, allowing opponents to assess their probability of winning without any need for social recognition or memory. For example, house sparrows (*Passer domesticus*) display a variably sized bib patch ([7]; though see [8]), great tits (*Parus major*) have a prominent black breast-stripe that varies in width ([9]; but see [10]), and some wasp species display their quality in their facial patterning [11]. The second main mechanism to decrease the cost of contents is the formation of relatively stable dominance hierarchies, which are primarily shaped by memory of repeated interactions and potentially by observations of the interactions among others [12]. Hierarchies can either be based entirely on social recognition and memory [13,14], or a combination of social recognition and some intrinsic and predictable markers (e.g. age or sex [15]).

Within stable groups, dominance hierarchies will allow the most precise assessment of the status of group co-members [16]. Conversely, in open societies, a badge-of-status provides an assessment mechanism that, while not as accurate, does not require individual recognition [17]. However, there is now increasing evidence that many species maintain complex differentiated social networks in which groups exhibit fission–fusion dynamics, or interact with individuals from other groups [18–20]. For example, many corvids and parrots exhibit classic communal roosts, where individuals sleep at stable roosts with potentially hundreds of individuals, forage in smaller fission–fusion subgroups, and occasionally also interact with members of other neighbouring roosts [21,22]. There have been various arguments for what strategies individuals may use to mediate aggressive interactions under these circumstances. First, the ability to segregate into sub-groups with flexible membership may decrease the cost of competition to the extent that the need for stable hierarchies or a badge-of-status is weakened [23] or absent [24]. Alternatively, complex social cognition may allow individuals to remember large numbers of individuals and interactions to maintain hierarchies (as argued for ravens [25]). A third understudied possibility is that individuals could use mixed strategies, with memory-based dominance hierarchies determining interactions among familiar individuals (e.g. at the roost-, or group-level), and with individuals either (i) deploying no assessment strategies towards relative strangers, (ii) assessing strangers by using a badge-of-status, or (iii) assessing strangers using a set of cues to their state. The potential existence of mixed strategies could help explain how animals manage the cognitive load associated with living in large-scale societies.

In Sheehan & Bergman [17], for example, the authors suggested that, after dispersal, juveniles should rely on quality signals when interacting with unfamiliar individuals, but once juveniles are settled, interactions reoccur, which should favour social recognition over quality signals. Empirical evidence of mixed strategies was provided by two recent studies investigating the potential for context-dependent use of badge-of-status in captive birds [26–28]. By experimentally manipulating colourful crown patches in blue tits (*Cyanistes caeruleus*) and golden-crowned sparrows (*Zonotrichia atricapilla*) and then inducing contests, the authors found that the crown patch size mediated contest outcomes among strangers, but was less important in determining contest outcomes in birds caught in the same area [26–28]. These results suggest that animals may use individual recognition to mediate interactions with familiar individuals, and status symbols in interactions with relative strangers [28]. However, these studies inferred familiarity did not explore whether individuals formed dominance hierarchies within familiar flocks. To the best of our knowledge, such mixed strategies are yet to be reported in natural interactions, or observed in the wild.

**Figure 1.**
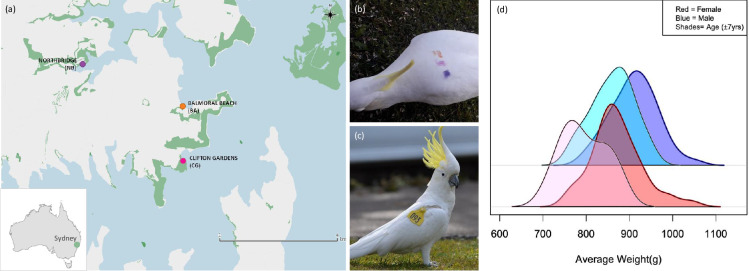
Study locations and marked birds and their respective weights. (a) Map of the three roosting communities included in the study. (b) Example of marking with temporary dye, violet pink orange—horizontal, that remains visible for 3−4 months. (c) Example of wing-tagged bird—093 Albie. (d) Distribution of average weights of individuals in grams, across age and sex classes. Dark blue are adult males (>7 years, *n* = 137), light blue are juvenile males (<7 years, *n* = 49), dark red are adult females (>7 years, *n* = 116) and pink are juvenile females (<7 years, *n* = 30).

Here, we investigate patterns of aggressive interactions within and between three neighbouring roost groups of wild sulphur-crested cockatoos (*C. galerita*). Sulphur-crested cockatoos (SC-cockatoos) are large, white, sexually monomorphic parrots that exhibit no clear plumage variation between individuals that might represent a badge-of-status (figure 1). Like many parrots, SC-cockatoos form year-round communal roosts of 100−1000 birds [22,29]. Within these roosts, SC-cockatoos display highly fluid fission–fusion dynamics, with flock size varying between 2 and 500 individuals [30,31]. In addition, birds also regularly forage with individuals of different roosts and occasionally engage in between-roost movements [22,29]. Despite these larger dynamics, individuals maintain stable, long-term relationships beyond the pair bond that are strongly suggestive of social recognition, including with birds from other roosts [22,29]. Our previous work has also shown that SC-cockatoos maintain dominance hierarchies at the level of the roosting community, with these hierarchies being stable over extended periods of at least 3 years, and likely to be memory based [32]. Given this social structure, we hypothesized that individuals would use mixed strategies; using dominance hierarchies to determine interaction decisions at the roost level, but deploying either no assessment strategies or cue-based assessment when interacting with individuals beyond this social group.

## 2. Methods

### 2.1. Study population

The study was conducted in an urban population of SC-cockatoos in Sydney, Australia, an area within the native range of the species. SC-cockatoos in this area use communal sleeping roosts of up to 200 individuals. These roosts are maintained year-round and likely over many decades, and consist of non-breeding and breeding birds, with breeding individuals nesting in tree-hollows in close proximity to the main roost [22]. In this area, roosts tend to be located 1.5−5 km apart, with birds generally foraging in an approximate 3 km radius around the roost [22], and returning to the roost regularly throughout the day. Our study was focused on three similarly sized neighbouring roost-sites in north Sydney, located at Balmoral Beach (BA), Clifton Gardens (CG) and Northbridge (NB): figure 1a, electronic supplementary material, table S1.

At each of the three roost sites, birds were first habituated to the researchers and then marked using non-toxic dye (Marabu Fashion Spray, MARABU GmbH) using methods detailed in Penndorf *et al*. [29]. Each individual was marked with a unique combination of one to four coloured dots, applied with sponges on the middle of the back (figure 1b). As part of a parallel project run at the same time, birds were also habituated and marked at two roost-sites outside of the focal study area (electronic supplementary material, table S1), in Manly (MA) and the Royal Botanic Gardens (BG; electronic supplementary material, table S1). In addition to the paint-marked birds, 144 birds equipped with wingtags as part of the ongoing citizen science study *Big City Birds* ([22,33], figure 1c) regularly visited the three roosting locations.

Overall, 411 individually identifiable birds were included in the study across the three roost locations, which we estimate to be 95% (CI = 0.92–0.98) of the individuals at each site (electronic supplementary material, table S1). Of these 411 birds, 373 were paint-marked, 28 were wing-tagged and 11 had distinctive physical features that meant marking was not required (e.g. healed injuries): electronic supplementary material, table S1. Age (juveniles: <7 years, adults: >7 years) and sex of birds were assessed by eye-colour [34]. Additionally, feathers were collected for a parallel study, and molecular sexing was used to sex juveniles (BA: *n* = 68, CG: *n* = 55, NB: *n* = 41, Penndorf *et al*. [29]). Finally, we recorded the weight of individuals by training them to step on a flat scale that read at 1 g accuracy in exchange for a food reward (e.g. sunflower seeds). This resulted in 214 birds being weighed 1–17 times each (mean: 4.3) over the 4 months across the three primary roost-sites (BA, CG and NB). Weight was highly repeatable within individual (0.78, 95% CI = 0.72–0.82, R-package *rptR* [35], number of bootstraps = 1000) and ranged from 717 to 1054 g. Males tended to weigh more than females, and adults more than juveniles (figure 1c).

### 2.2. Social data collection

We recorded social associations and interactions over two 10 day periods from 8 to 20 July and 19 September to 2 October 2019. Behavioural observations were collected daily for 3 h (July) or 2.5 h (September), during which periods birds were encouraged to forage on the ground by scattering small amounts of sunflower seeds over an approximate 385−500 ${\text{m}}^{2}$ area of grass close to the roost location (300−680 m distance).

During each sampling period, presence scans [36] were conducted every 10 min to record the identity of all birds present. We defined present as being identifiable within the park. Additionally, we used all occurrence sampling [36] to record aggressive interactions, recording the time, the identities of the individuals involved, as well as the sequence of the interaction. Aggressive interactions within a dyad were considered independent if there were more than 60 s between two aggressive events involving the same two individuals. If different dyads interacted very close in time, we prioritized recording the identities of winners and losers omitting the sequence.

### 2.3. Dominance hierarchies

Given that the hierarchies of both observation periods were highly correlated [32], we combined the interaction data from both observation periods. We then calculated a separate dominance hierarchy for each of the three roost-sites using randomized Elo-ratings (R-package *aniDom* [37], sigma = 1/300, *K* = 200, randomizations = 10 000) and included only individuals with 10 or more agonistic interactions at a given roost-site location (BA: $N_{ind}$ = 126; CG: $N_{ind}$ = 93 and NB: $N_{ind}$ = 74; [37]).

To test whether rank was correlated with the intrinsic factors of age, sex and weight, we ran a generalized linear mixed model using the R-package *lme4* [38]. We tested whether standardized rank was predicted by the average weight of an individual, as well as age and sex. Since individuals could appear in the hierarchies of several sites (26 individuals present in two hierarchies, two individuals present in three hierarchies), we also included site and individual ID as random variables.

### 2.4. Social decision-making

Decisions about interactions were made at two levels. First, an individual (hereafter *initiator*) decides whether to engage in an aggressive interaction. Second, the individual that is being aggressed (hereafter *receiver*) chooses whether to retreat or reciprocate. Decision-making in both cases is based either on an assessment of the opponent’s resource-holding potential (RHP) [39] or on memory, including prior experience or knowledge of relative rank [28].

We hypothesized that in the absence of local knowledge, individuals would use assessment of RHP to decide on whether or not to engage in an aggressive interaction. We further hypothesized that when individuals had knowledge about the social environment, they would instead base their decision-making on the dominance hierarchy. In order to test these hypotheses, we considered two instances of decision making: the decision of the initiator about whom to aggress, and the decision of the receiver about how to react to the aggression.

As aggressive interactions involving females were relatively rare, we restricted interactions to male–male dyads only. As a proxy for RHP, we used an individual’s average weight, which is likely to be correlated to overall body size. To estimate the familiarity, we made two assumptions. First, since we only recorded interactions for a limited time each day, we considered the location—or social environment—as proxy for the likelihood that individuals would know each other. Second, to classify the familiarity of individuals with a social environment, we considered individuals familiar with the social environment at the site if they were present in at least 5% of the scans conducted at the site (determined through break-point analysis, [40] electronic supplementary material, section S1). Otherwise, individuals present in fewer than 5% of scans at the site were considered unfamiliar with the social environment.

#### 2.4.1. Deciding whom to aggress

To test which cues SC-cockatoos are most likely to base their decisions to initiate an aggressive interaction upon, we used a two-step approach. First, we tested the influence of weight on decision-making. For this, the data were divided into two groups: (i) interactions where the initiator had no social knowledge, and (ii) interactions where the initiator had social knowledge. In the second step, we focused only on interactions where the initiator had social knowledge to test whether familiar individuals use an alternative, rank-based, interaction strategy.

In order to test the strategic use of aggressions for each of the above-described scenarios, we adapted the method developed by [41] and modified by [3], consisting of several steps. First, we calculated the difference between individuals in weight or rank as:

— (weight initiator) − (weight receiver) for the analysis on weight
— (rank initiator) – (rank receiver) for the analysis on rank, where dominance ranks were calculated using randomized elo-rating (R-package *aniDom* [37]; sigma: 1/300, *K*: 200, randomizations: 10 000).

The interaction data were used to count the number of observed directed interactions across each pairwise combination of individuals. To generate expected interaction patterns, we implemented a permutation procedure, where each iteration (*n* = 1000) randomly selected one interaction and assigned it to be either used to calculate the dominance hierarchy, or to be used in the assessment of the interaction strategy. If the interaction was selected for the assessment of the interaction strategy, a new receiver of the interaction was randomly selected among all individuals present (i.e. individuals present in the scan just before and just after the interaction, including the original receiver but not the initiator). Permutations were limited to interactions where at least three individuals were present at the site at the time of the interaction (including the original initiator and receiver).

We ran 10 000 permutations before calculating the difference between observed and permuted datasets. If the observed tendency to interact follows a random pattern, the confidence intervals should overlap zero. A positive or negative value that does not overlap zero indicates that interactions between individuals of that rank difference occurred more or less often than expected by chance. As interaction strategies towards individuals positioned higher or lower than oneself in the hierarchy may differ [3], we fitted separate smoothing splines for positive and negative rank differences (*smooth.spline* R-function, d.f. = 3, lambda = 0.04).

#### 2.4.2. Analysis of responses to aggression

To examine the decision of the receiver, we identified individuals’ responses to aggressions. To do so, we classified all interactions with recorded sequences having received an aggressive response or not, depending on whether the receiver of the initial aggression responded aggressively towards the initiator of the interaction (response—1) or not (retreat without aggressive display—0). These were defined as interaction sequences in which the receiver responded aggressively towards the initiator of the interaction, independent of who eventually won the interaction. Across all roost sites, this resulted in 3845 interactions between 396 individuals (electronic supplementary material, table S1).

We first tested the influence of weight and familiarity on the likelihood of an aggressive response when being aggressed. To do so, we modelled the response of aggressive interactions (binary, 0: the receiver retreated without showing any aggression, 1: the receiver aggressed the initiator), as function of the absolute weight difference of the dyad (in grams), the knowledge status of both receiver and initiator (familiar/unfamiliar), the relative weight (binary, whether the initiator was heavier than the receiver or not), as well as the interactions between all terms.

In the second step, we focused only on a subset of interactions, where both individuals were knowledgeable of the social environment to test the influence of rank difference on the likelihood of aggressive response. Therefore, we tested whether aggressive responses were predicted by the absolute rank difference in the knowledge status of both receiver and initiator, whether the initiator was of higher/lower rank than the receiver, as well as the interactions between all terms.

For both analyses, we constructed binomial GLMMs (package *lme4* [38]) that included the base predictor for each hypothesis (absolute weight or rank differences) and all possible combinations of additional predictors (familiarity and whether the initiator was heavier for the weight hypothesis, and whether the initiator was heavier or higher in rank for the rank hypothesis, table 1) that could contribute to an aggressive response. We then compared models using AIC to identify which set of additional predictors were important in modulating the role of the hypothesized heuristic.

**Table 1.** Predictors of the receiver’s response to being aggressed. This response was encoded as 0 (the receiver retreated without displaying an aggressive response) or 1 (the receiver acted aggressively towards the initiator).

model	predictors
aggressive response as a function of weight difference unfamiliar and familiar individuals only	— familiarity (binary, T/F) — absolute weight difference (continuous, grams) — initiator heavier (binary, T/F)
response to aggressions as a function of weight and rank differences familiar individuals only	— absolute weight difference (continuous, grams) — absolute rank difference — initiator heavier (binary, T/F)

## 3. Results

The roost-level hierarchies included between 74 and 126 individuals (i.e. birds with ≥10 observed interactions at a specific location; electronic supplementary material, table S1). From these 265 individuals, we had known weights for 176. We recorded 6402 aggressive interactions across all birds at each of the three roost sites, of which 5447 were between individuals in the same dominance hierarchy (*n* = 265 birds). Of these, 3859 observations had a full sequence starting with information about the initiator and receiver (electronic supplementary material, table S1 for a subset of birds per site).

### 3.1. Predictors of dominance rank

Overall, standardized weight was not significantly correlated with rank (β = −0.0002; *p* = 0.570). When repeating the same analysis within males only, we found again that weight did not predict dominance rank (β = −0.0005, *p* = 0.63). However, as expected, males tended to be more dominant than females. Given this, and given that most interactions involved males (46% of interactions involving individuals of known sex), the subsequent analyses were conducted only within males.

### 3.2. Social decision-making

#### 3.2.1. Initiation of interactions

**Figure 2.**
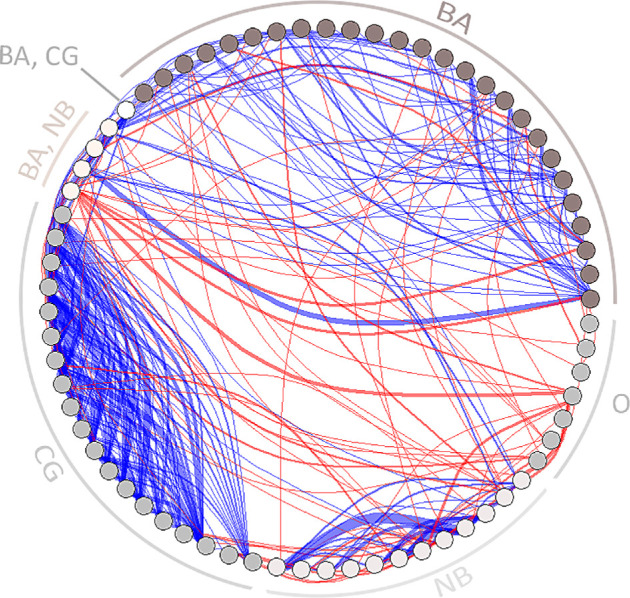
Aggressive interactions between males. Nodes are shaded by the familiarity of the individual with specific social environment(s), indicated as label for each node (BA, CG and NB). Individuals that were transient to all three sites are marked as other (O) roost-membership. Edges are coloured depending on the familiarity with the social environment. Blue edges represent interactions between familiar individuals. Red edges represent interactions between individuals from different social environments.

If the initiator was not knowledgeable of the social environment, they preferentially targeted other individuals close in weight (−170 to 200 g; electronic supplementary material, figure S1a). In contrast, interactions initiated by knowledgeable individuals (present in >5% of the scans at that specific location) showed no significant influence of weight on the decision to initiate an aggressive interaction, though there was a tendency of individuals to initiate interactions with others that were slightly lighter (<80 g, electronic supplementary material, figure S1b). Rather, when individuals were knowledgeable of the social environment, relative rank difference was a better predictor of social interactions. In this case, individuals directed aggressive interactions towards lower-ranking individuals while avoiding aggressing those higher in rank (figures 2 and electronic supplementary material, S1c).

#### 3.2.2. Responses to aggressions

**Figure 3.**
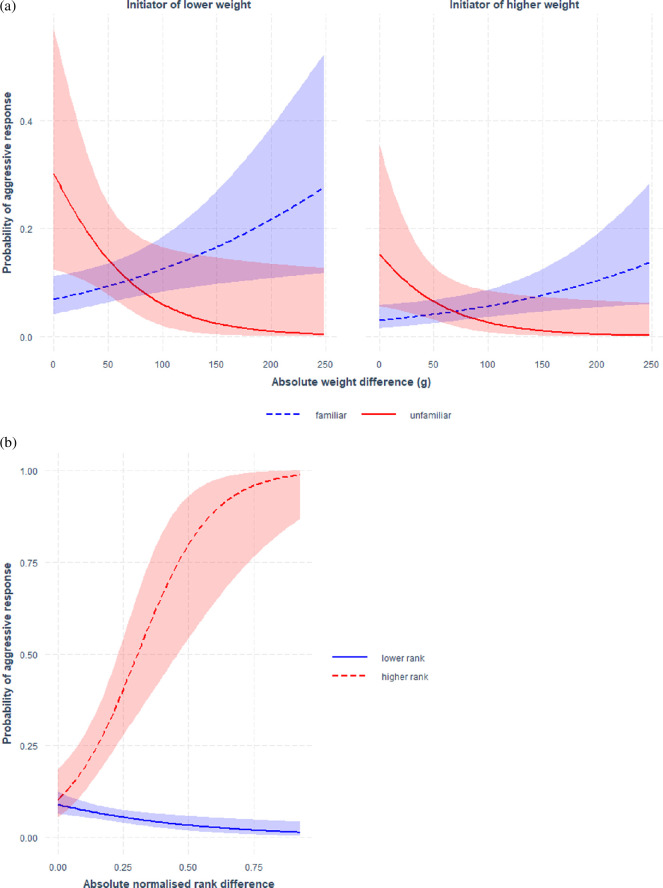
Probability of responding aggressively towards the initiator depending on differences in weight and rank, and whether birds were familiar or not. (a) We found that aggressive responses were generally more likely if the initiator had a lower weight than the receiver (dashed lines versus solid lines). In the absence of social knowledge (red lines; unfamiliar), we further found that aggressive responses were more likely if the two individuals were closer in weight. (b) Aggressive responses in interactions between knowledgeable individuals were only likely to occur if the initiator was lower in rank (blue line), with this probability increasing as this difference in rank increased. Line colours are labelled from the perspective of the initiator.

The model of responses to aggressions most supported by the data included an interaction between absolute weight difference and familiarity, plus a term capturing whether the initiator was higher or lower in weight. This suggests that familiar and unfamiliar individuals had different tendencies to respond to aggressions for a given difference in weight. Specifically, we found that aggressive responses in our dataset were best predicted by an interaction between weight difference and familiarity, and whether the initiator was heavier than the receiver of the interaction (figure 3a,b, electronic supplementary material, table S2). That is, if one individual in the dyad had no social knowledge, then it was much more likely to respond aggressively if individuals were close in weight (e.s. = 0.03, s.e. = 0.01, *p* = 0.05). This probability was also higher if the receiver was heavier than the initiator (e.s. = −0.88, s.e. = 0.37, *p* = 0.02—figure 3a,b). If both individuals were familiar with the social environment, aggressive responses were also more likely if the receiver was heavier, independent of the weight difference (e.s. = −1.77, s.e. = 0.81, *p* = 0.03). There was a possible trend towards a higher probability of aggressive responses when the weight difference was large (figure 3a,b); however, this was entirely driven by two interactions between the same dyad.

Second, we focused on a subset of the data containing only interactions between individuals familiar with the social environment to test whether rank differences predict responses to aggressive interactions, electronic supplementary material, table S3. For the effect of rank difference, the model most supported by the data included absolute rank difference (e.s. = 4.12, s.e. = 0.97, *p* < 0.001), as well as an interaction between absolute rank difference and whether the initiator was ranked higher than the receiver (e.s. = −6.92, s.e. = 1.12, *p* < 0.001). Specifically, we found that if the initiator was ranked higher, aggressive responses only occurred if individuals were close in rank (figure 3c). If, on the other hand, the initiator was ranked below the receiver in the hierarchy, the probability of aggressive responses was high and increased with increasing rank difference (figure 3c). While absolute weight difference was included in the best model, the effect was insignificant (e.s. = 0.002, s.e. = 0.002, *p* = 0.47).

## 4. Discussion

We found that SC-cockatoos used mixed decision-making strategies in their aggressive interactions. First, for dyadic interactions where at least one individual was not knowledgeable of the social environment (e.g. an individual joining a foraging flock at a different roost site), individuals disproportionately directed aggression towards those close above and below themselves in weight, peaking at 90 g heavier. For a male of average weight (900 g), this difference represents 10% of body weight, a difference that may not be ascertainable visually, suggesting that individuals are challenging opponents of similar overall body size. Within these interactions, the probability of responding aggressively towards the initiator also depended on whether the individuals were close in weight, with this probability being higher if the receiver was higher in weight. This contrasted the interactions among familiar individuals. Here, weight had little correspondence with whom individuals directed aggression towards and only affected the probability of aggressive responses when the initiator was considerably lower in weight than their aggressor.

Second, within regularly interacting individuals, we found that individuals were less likely to direct aggression towards those above them in the hierarchy than expected by chance and were more likely to direct aggression towards those below them, with most aggressions occurring at 15–45% rank difference. Response to received aggression was then only likely to occur when the receiver was much higher ranked than their aggressor. Together with our results on interactions between less familiar individuals, these results provide evidence that SC-cockatoos are engaging in a mixed strategy that depends on the familiarity between interacting individuals. When facing less familiar individuals, decisions to interact—or respond—were based on the relative weight difference. Conversely, social decisions between familiar individuals were based on social recognition and memory, with individuals generally aware of relative rank differences between themselves and familiar others.

### 4.1. Interactions with strangers: using proxies for resource-holding potential

When interacting with non-residents, SC-cockatoos used body size (weight) in their aggressive decision-making. This suggests that (i) there are limits to social cognition in SC-cockatoos, with individuals unable to retain (or not using) memory of relative rank for individuals outside of their group, despite being members of nearby roosts and therefore potentially having interacted previously to our observation period, and (ii) SC-cockatoos are assessing these individuals using a set of cues to their state, including sex and body size. This finding contributes to the growing evidence of mixed strategies in systems with fission–fusion dynamics [26–28], but extends this body of work in two important ways. First, it demonstrates the existence of such mixed strategies in wild, naturally interacting individuals, and where stable, quasi-linear dominance hierarchies are maintained within familiar individuals. Second, it gives evidence for the existence of such strategies even in the absence of clear status signals. This is particularly intriguing, as it has been previously suggested that the use of social recognition could constrain the evolution of open social systems, while the use of quality signals may facilitate the evolution of open social systems [17]. It further suggests that social cognition cannot be assumed in systems without such signals of quality.

While we show an effect of assessment of body size (as estimated by weight) on decision-making, we cannot exclude the possibility that weight is correlated to a yet undescribed status signal in this species. One possibility could be crest length or colour, given its importance for signalling across Cacatuidae [42,43], or vocalizations [44,45]. Another potential status signal might be UV-reflective plumage [46,47]. Such fluorescent patches are found in a majority of parrot species (140 out of 143 species examined by [48]). SC-cockatoos have yellow feathers on their cheek, under-wing and crests that exhibit ultraviolet fluorescence (figure 1b) [48]. The function of this fluorescence in SC-cockatoos has not been studied. However, UV-reflective plumage is used to inform mate choice in Budgerigars [46], and it is largely thought to be a sexual signal [47,49]. Whether it could also signal status and/or condition in parrots warrants further investigation, as previous research argued that secondary sexual characters can also serve as honest signals of RHP [50]. That said, while more targeted research is warranted to test whether the yellow colouration of SC-cockatoos could serve as an honest signal of RHP, our current data seems to suggest that this is unlikely to be the primary cue or signal. We observed no obvious movement or display of cheek patches during aggressive interactions. Crest movements are often induced by surprising or alarming stimuli [42], and are utilized in breeding displays at nest hollows (personal observation). These observations are further strengthened by our data, as only 16% of all aggressive interactions included crest-erection (15% of interactions involving familiar individuals, and 19% of interactions involving unfamiliar individuals).

### 4.2. Interactions with familiar individuals: relying on social cognition instead of proxies for rank

Our results from interactions between relative strangers suggest that SC-cockatoos are able to assess body size (for which we used weight as a proxy), and perhaps use this as a signal of RHP. Yet within roosts, weight did not determine current dominance rank, although we cannot exclude that it could have a role in initial establishment of rank. This may seem surprising, given that (i) flocks of wild SC-cockatoos have been previously suggested to be too large to form effective dominance hierarchies [30]—but see [32], (ii) the high degree of fission–fusion dynamics in this species means individuals may encounter hundreds of others over days and weeks [22,29], and (iii) weight does partly influence decisions to initiate and/or respond to aggressive interactions. One potential explanation is that weight-based hierarchies tend to be restricted in terms of maximum group size when weight differences become too small to assess [51]. Groups of SC-cockatoos may therefore be too large for primarily weight-based hierarchies to be useful. Whether similar limitations exist for potential badges of status, such as for example, UV signals, has yet to be explored. In contrast, SC-cockatoos must have the cognitive ability to learn and remember their position in the dominance hierarchy, noting that this hierarchy is highly stable over time [32]. Previous work has suggested that SC-cockatoos exhibit preferred social associates [29], and form stable long-term relationships [22]. This supports other work in other parrot species that is also suggestive of extensive individual recognition [52,53]. Overall, this suggests that SC-cockatoos may possess sufficient memory of past interactions to allow them to track dominance relationships in large social groups [54]. Given that memory-based dominance hierarchies are predicted to be the most reliable way to assess status, this would lead individuals to rely on social cognition within roosts, only using cues to body condition when this is not possible due to complete unfamiliarity or cognitive constraints (in the case of rare interaction partners).

## 5. Conclusions

Our previous work has shown that wild SC-cockatoos form stable long-term relationships [22] and maintain some social relationships even after dispersal into different roost groups [29]. This suggests that SC-cockatoos possess extensive memory of past interactions—an ability displayed by other large-brained bird species that exhibit fission–fusion dynamics, such as common ravens (*Corvus corax* [25]). This social cognition is further exhibited within roost groups, with birds maintaining impressively large dominance hierarchies that are stable over time [32]. However, when this knowledge is lacking (i.e. when facing less familiar individuals), aggressive decisions in SC-cockatoos to interact—or respond—are based on the relative weight difference. This implies that, even with good memory and no signals of quality to use as proxy for RHP, a mixed strategy that depends on the frequency of aggressive encounters might be more adaptive in open societies than a sole reliance on social recognition and memory, perhaps because it may reduce errors from inaccurate recollection or outdated information. Taken together, our results suggest that social knowledge remains an important determinant of aggressive interactions, even under such high fission–fusion dynamics, but that individuals can flexibly incorporate other potential cues of competitive ability when recent knowledge is lacking.

## Data Availability

The data are available on Dryad [55]. Supplementary material is available online [56].
